# Machine learning reveals orbital interaction in materials

**DOI:** 10.1080/14686996.2017.1378060

**Published:** 2017-10-26

**Authors:** Tien Lam Pham, Hiori Kino, Kiyoyuki Terakura, Takashi Miyake, Koji Tsuda, Ichigaku Takigawa, Hieu Chi Dam

**Affiliations:** ^a^ Japan Advanced Institute of Science and Technology, Nomi, Japan.; ^b^ Elements Strategy Initiative Center for Magnetic Materials, National Institute for Materials Science, Tsukuba, Japan.; ^c^ Center for Materials Research by Information Integration, Research and Services Division of Materials Data and Integrated System, National Institute for Materials Science, Tsukuba, Japan.; ^d^ Research Center for Computational Design of Advanced Functional Materials, National Institute of Advanced Industrial Science and Technology, Tsukuba, Japan.; ^e^ JST, PRESTO, Kawaguchi, Japan.; ^f^ Graduate School of Information Science and Technology, Hokkaido University, Sapporo, Japan.; ^g^ Department of Computational Biology and Medical Sciences, Graduate School of Frontier Sciences, University of Tokyo, Kashiwa, Japan.; ^h^ RIKEN Center for Advanced Intelligence Project, Tokyo, Japan.

**Keywords:** Material descriptor, machine learning, data mining, magnetic materials, material informatics

## Abstract

We propose a novel representation of materials named an ‘orbital-field matrix (OFM)’, which is based on the distribution of valence shell electrons. We demonstrate that this new representation can be highly useful in mining material data. Experimental investigation shows that the formation energies of crystalline materials, atomization energies of molecular materials, and local magnetic moments of the constituent atoms in bimetal alloys of lanthanide metal and transition-metal can be predicted with high accuracy using the OFM. Knowledge regarding the role of the coordination numbers of the transition-metal and lanthanide elements in determining the local magnetic moments of the transition-metal sites can be acquired directly from decision tree regression analyses using the OFM.

## Graphical Abstract

**Figure:**
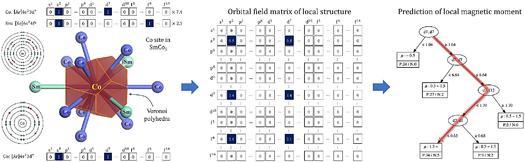

## 1. Introduction

Recently, the increasing volume of available experimental and quantum-computational material data, along with the development of machine learning techniques, has provided a new opportunity to develop methods for accelerating discoveries of new materials and physical and chemical phenomena. By using machine learning algorithms, hidden information on materials, including patterns, features, chemical rules, and physical laws, can be automatically discovered from both first- principles-calculated data and experimental data [1–8]. It is commonly known that, in a material dataset, the most important information for identifying a material is its structure. Information on the structure of a material is usually described using a set of atoms with their coordinates and periodic unit cell vectors, which are required for crystalline systems. From the viewpoint of data science, the material data using this primitive representation can be categorized as unstructured data, and the mathematical operations performed on such material data involve the algebra of sets only. Therefore, advanced quantitative machine learning algorithms cannot be applied directly and effectively to conventional material data, owing to the limitation of the algebraic operations of the primitive data representation. In order to apply well established machine learning methods, including predictive learning and descriptive learning, it is necessary to convert theprimitive representation into fixed-dimensional vectors or matrices, such that the comparison and calculations using the new representation reflect the nature of the materials and the actuating mechanisms of the chemical and physical phenomena. Various methods for encoding materials have been developed in the field of materials informatics.

Previously, Behler and coworkers [9–15] utilized atom-distribution-based symmetry functions to represent the local chemical environments of atoms with a cutoff radius of approximately 1.0 nm, and employed a multilayer neural network to map these chemical environments to the associated local (atomic) energies. The global (total) energy of a given material was then calculated by taking the summation of its local energies. This descriptor is recognized as one of the most successful descriptors for fitting the atomic potential energy surfaces. Bartók and colleagues [16–18] employed the atomic density distribution to compare molecules and solids. Gaussian kernels were used to smoothly approximate atomic density in a local structure. And the similarity between two local structures was estimated by overlapping of their atomic densities which are expanded by spherical harmonic functions. Another successful descriptor was developed by Rupp and coworkers, and is known as the Coulomb matrix (CM) [19–21]. The CM descriptor includes all the pairwise structural information on the atoms in a system and is long range with a length dependence of 1 / *r*. The CM is used for predicting the atomization energies of small isolated organic molecules and obtained very successful results [20]. Complementary to the mentioned descriptors, there is an effort of combining many types of materials representation including atomic information, the partial radial distribution function, the generalized radial distribution function, etc., together with their covariances, to predict cohesive energies with high accuracy [22]. In spite of the advantage in some predictive analyses, the above descriptors cannot be effectively employed to other interesting mining tasks that require high interpretability of the learning results, for instance, the problems regarding pattern detection of materials behaviors, the extraction of hidden chemical/physical knowledge from a material dataset, the visualization of material datasets in a low dimensional space, etc.

Another interesting attempt at descriptor design involves the introduction of information on the electronic structures. Previously, Isayev et al. used band structures and density of states (DOS) fingerprint vectors as representations of materials to visualize the material space [5]. However, use of information on the electronic structure requires first-principles calculations, which have a high computational cost. We believe that it is a good direction if we can take into account information of the electronic states, and the atomic electron configuration may be regarded as the zeroth order approximation and could be considered as a viable substitution. Structural fragment arrangement has also been utilized to encode materials in order to predict their physical properties [5,23]. This kind of descriptor exhibits good performance for molecular systems, and important fragment patterns concerning a certain material property can be discovered from the learned results. Through consideration of these descriptors, the present authors obtained the concept of developing a descriptor for crystalline materials based on a local structure comprised of a center atom and its neighboring atoms (this local structure can also be regarded as a structural fragment), along with information on the atomic electronic structure (electronic configuration) of the constituent atoms.

To render data-driven approaches meaningful and useful for materials science studies, it is necessary to design material representations with which the results derived using machine learning methods can be interpreted in the language of physical chemistry. It has been well established in fundamental chemistry that certain important aspects of the electronic structure can be deduced from a simple description of the nearest atoms or valence electrons around an atom in a molecular or crystalline system; e.g. the Lewis theory provides powerful tools for studying molecular structure [24]. The ligand field and crystal field theories are examples of other theories developed based on this intuition to classify or categorize local atomic environments, and several fruitful results have been obtained using these theories [25]. Needless to say, within these theories, information regarding the long-range interactions can be included by embedding the information on the local chemical environment of the nearest atoms using a *convolutional manner*. We utilize this heuristic intuition to implement the above-mentioned concept of developing a novel representation by incorporating the information on the local structure and the number of valence orbitals (electrons) coordinating the valence orbital of the center atom. We name this type of descriptor the ‘orbital field matrix (OFM)’.

In this work, with emphasis on the interpretability of the derived learning results, we design a material descriptor that (1) utilizes information on the local structure, (2) incorporates the valence atomic configuration, and (3) accepts algebraic operations to construct global descriptors from local descriptors. To verify the applicability of the proposed material representation, we focus on magnetic materials based on bimetal alloys of lanthanide metal and transition-metal (LAT) and LAT alloys including a light element X, which may be B, C, N, or O (LATX). We first examine the decision trees for predicting the magnetic moments of Mn, Fe, Co, and Ni in LAT alloys. The decision trees learned from the LAT alloy data show that the coordination numbers of the occupied *d* orbitals of the transition-metals and the occupied *f* orbitals of the lanthanides play important roles in determining the local magnetic moments of the transition-metal sites. The obtained results confirm the interpretability of our OFM representation regarding structural and physical chemistry. In addition, kernel ridge regression (KRR) analyses using standard techniques and similarity measures are implemented in learning prediction models to quantitatively predict the local magnetic moments of transition-metal sites in LAT alloys, formation energies for LATX materials, and atomization energies for organic molecules. Our computational experiments show that the OFM representation can accurately reproduce the local magnetic moments of transition-metal sites in LAT alloys, formation energies of crystalline systems, and atomization energies of molecular systems. The high prediction accuracy confirms the practicability of our OFM representation.

## 2. Methodology

### 2.1. Representation of materials

**Figure 1.**
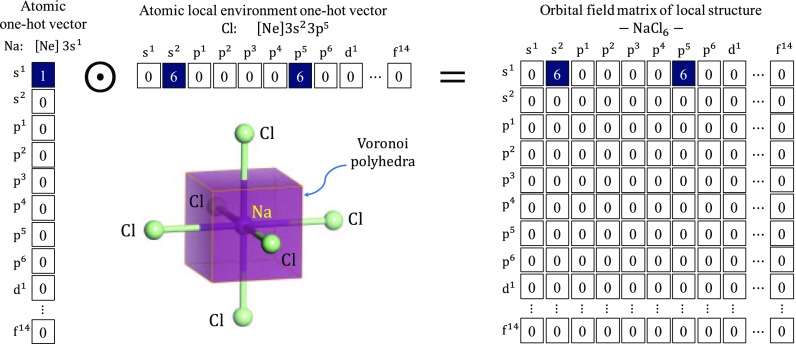
OFM representation for an Na atom in a regular octahedral site surrounded by six Cl atoms: atomic one-hot vector for Na (left), representation for the six Cl atoms surrounding the Na atom (middle), and representation for the Na atom surrounded by six Cl atoms (right).

To design the representation for a material, we start with the representation for an atom as a material building block. We utilize the standard notation for electron configuration to develop the representation for an atom; e.g. the electron configurations of Na and Cl are [Ne]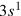 and [Ne]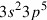, respectively. In order to convert this standard notation into a numerical vector, we borrow the concept of one-hot vector in the field of natural language processing, in which a word is represented by a bit vector having the dimension of the number of words in a dictionary. The vector consists of elements with values of 0, with the exception of a single element used uniquely to identify the word. The representation of an atom is then converted from the standard notation into a one-hot vector 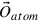 by using a dictionary comprised of the valence subshell orbitals: 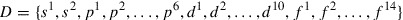 (e.g. 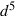 indicates the electron configuration in which the atomic valence *d* orbital holds five electrons), which consists of 32 elements (Figure 1).

Next, we design the representation of the coordination number. It is not easy to define the coordination number for realistic crystal structures and there exist a number of such definitions. In this study, we adopt the definition by O’Keeffe [26], which utilizes the solid angles determined by the faces of the Voronoi polyhedra. This method can give the same coordination numbers for the high-symmetry atomic environment and evaluate coordination numbers for the lower-symmetry atomic environment automatically and with no ambiguity. We implement this method using Python Materials Genomics (pymatgen) code [27].

We represent a local structure surrounding an atom by considering the sum of the weighted vector representations of all surrounding atoms in the local structure using 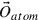 and the coordination number. A central atom at site *p* in a local structure can be represented using the OFM with the elements 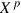, which are defined as follows:(1)
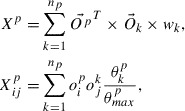



where 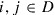; *k* is the index of the nearest-neighbor atoms; 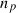 is the number of nearest-neighbor atoms surrounding site *p*; 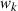 is a weight that represents the contribution of atom *k* to the coordination number of the center atom, *p*; 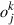 and 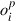 are elements of the one-hot vectors of the *k*th neighboring atom and *p* (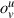 is 1 if the valence orbitals of the atom at site *u* have electron configuration of type *v*; otherwise, it is 0) representing the electron configuration. Further, 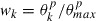, gives a weight of atom *k* in the coordination of the central atom at site *p*, where 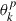 is the solid angle determined by the face of the Voronoi polyhedron separating *k* and *p*, and 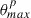 is the maximum among 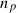 of them. An element of OFM, 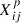, represents the number of orbitals *j* coordinating the center orbital *i*.

Additionally, to incorporate the information on the sizes of the valence orbitals, the distance 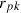 between *p* and *k* should be included in 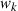. We propose the following form for the calculation of the OFM elements:(2)
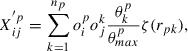



where 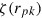 is a function representing the contribution of 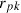 to 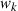. In this work, we use the inverse of the distance as the distance-dependent weight function: 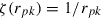. We use this 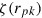 to distinguish atoms of the same valence configuration with different core shells and to describe the length dependence between the atoms. (Note that we can add the information on the core shells to the hot vector without losing the algebraic operation.)

Composing the descriptor for a structure (a molecule or a crystal system) from its local structure representation requires careful consideration. From the data science viewpoint, the composed descriptors should include as much information as possible. On the other hand, from the materials science viewpoint, the descriptors should be composed so that the natures of the target physical properties are reflected appropriately. For simplicity, in this work, for the atomization energy of a molecule (which is proportional to the molecule size), we take the sum of the descriptors of the local structures as the descriptor for the entire structure:(3)
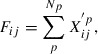



where *F* is the OFM representing the entire molecule. For the formation energy (per atom) of a crystal, which is not proportional to the system size, the descriptor for the entire structure is obtained by averaging the descriptors of the local structures:(4)
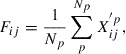



where 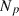 is the number of atoms in the unit cell.

## 3. Results and discussion

### 3.1. Prediction of local atomic properties

We now examine how the OFM can be employed to predict the local atomic properties of materials. In this work, we focus on the local magnetic moments of transition-metals in LAT alloys (in ferromagnetic configuration), the dataset of which includes 658 structures collected from the Materials Project database [28,29]. We select the structures by combining transition-metals and lanthanides from the sets of 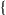
Sc, Ti, V, Cr, Mn, Fe, Co, Ni, Cu, Zn, Y, Zr, Nb, Mo, Tc, Ru, Rh, Pd, Ag, Cd, Hf, Ta, W, Re, Os, Ir, Pt, Au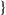 and 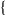
La, Ce, Pr, Nd, Pm, Sm, Eu, Gd, Tb, Dy, Ho, Er, Tm, Yb, Lu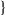. We employ Vienna Ab Initio Simulation Package (VASP) 5.4.1 [30–33] with the generalized gradient approximation (GGA)/Perdew-Burke-Ernzerhof (PBE) exchange-correlation functional[34,35] to calculate the local magnetic moments of these structures. We followed the Materials Project database regarding the selection of projector augmented wave (PAW)projectors [36,37], and employed pymatgen 4.3.0 [27] to prepare the VASP input files with 0.1eV Gaussian smearing of MITRelaxSet and a k-point mesh density of 150 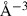. The energy cutoff is 520 eV. The VASP-PAW includes scalar relativistic effects by default. We perform collinear spin calculations without spin-orbit coupling. The systematic simulations performed in this study were conducted with the assistance of the Organizing Assistant for Comprehensive and Interactive Simulations (OACIS) [38].

In LAT alloys, three types of exchange interactions exist, including the exchange interaction between transition-metal (T) atoms in the T sub-lattices (T– interaction), the exchange interaction between lanthanide metal (LA) atoms and the T sub-lattices (LA –T interaction), and the exchange interaction between lanthanide metal atoms in the LA sub-lattices (LA – interaction). The exchange interactions involving LA elements are mediated by their 5*d* states, because of the strong spatial localization of the 4*f* states. The LA–T interaction is weak and the LA–LA interaction is marginal, in comparison to the T–T interaction. Our description of the local structure in terms of the coordination of the valence electrons is expected to include a significant amount of information regarding these magnetic interactions, which are essential for predicting the local magnetic moment. We first examine which elements in the OFM determine the local magnetic moments of the Mn, Fe, Co, and Ni sites in the LAT dataset through decision tree regression analyses.

**Figure 2.**
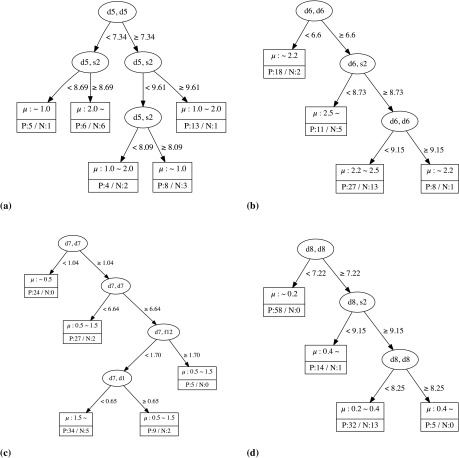
Decision tree regression for Mn (a), Fe (b), Co (c), and Ni (d). In each leaf, the upper part indicates the values of the local magnetic moments, whereas the lower part indicates the number of positive (P) and negative (N) examples.

To obtain the coordination information, we first employ Equation (1) to analyze the local magnetic moments, without considering the effects of different atomic orbitals having the same angular quantum numbers, but different principle quantum numbers. Weabbreviate 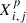 to 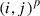, which represents the number of orbitals *j* surrounding orbital *i*. For instance, to encode the local structures of the metal sites in Sm-Fe alloys via OFM, we begin by representing the valence electron configuration of atomic Fe by 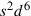 ([Ar] 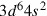) and that of Sm by 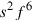 ([Xe] 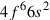). The 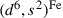 element in the derived OFM indicates the total coordination number of the Fe sites, as 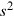 appears in both the Fe and Sm sites. The 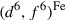 element represents the number of Sm sites surrounding the Fe sites, and the number of Fe surrounding an Fe site can be found at the 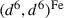 element. For simplicity, we drop the superscript (*p*) hereafter. The decision tree regressions for the local magnetic moments of the Mn, Fe, Co, and Ni sites derived from the data are summarized in Figure 2. It is clearly apparent that the (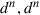) elements dominate the decision trees while the (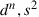), (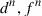), or (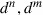) elements decorate the trees. This is consistent with the fact that the local magnetic moment of a transition-metal site is determined mainly by the number of unpaired electrons of the *d*-orbitals of the central transition-metal atom as well as by the T–T interaction between the same element due to the energy level relation. The appearance of (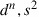), (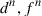), or (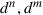) elements indicates that the LA–T interaction also plays a significant role in the determination of the local magnetic moment.

The tree for the Fe site cases shows that the magnetic moment is less than 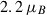 when the 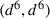 element is less than 6.6 or greater than 9.15. This result implies that the Fe atom appears to have a smaller magnetic moment when surrounded by less than seven Fe atoms or more than nine Fe atoms. The latter case reminds us of the anti-ferromagnetic ground state of face-centered cubic (fcc) Fe with 12 as 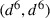. Further, the magnetic moments of the Fe sites may be greater than 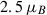 when the 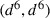 element is greater than 6.6, but the 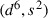 element (namely, the total coordination number including the contribution of the lanthanide metal atoms) is less than 8.73. The decision tree for the Ni sites shows that those sites tend to have a small magnetic moment (less than 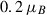) when the 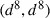 element is less than 7.22. However, a large magnetic moment (greater than 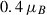) can be obtained when the 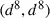 element is greater than 8.25. This implies that the Ni atom has a large magnetic moment when surrounded by more than nine Ni atoms. The magnetic moments of the Ni sites may be greater than 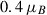 when the 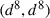 element is greater than 7.22, but the 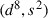 element (namely, the total coordination number including the contribution of the lanthanide metal atoms), is less than 9.15. This result is also qualitatively consistent with the observation that Ni cannot sustain its magnetic moment alone in metals [39].

For the Co sites, we see that the decision tree uses (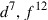) and (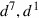), where 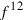 comes from Er and 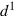 comes from La, Ce, Gd, and Lu at the lower branches. Careful analysis reveals that these branches are constructed to separate the cases of 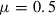
–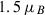 for LA-Er and (La, Ce, Gd, Lu)-Co from the case of 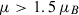. There are five Er-Co with a local magnetic moment of 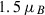 and five Er-Co with a local magnetic moment of less than 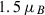, the criterion of which is (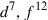) = 1.7. The (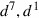) leaf separates Ce-Co and La-Co (the magnetic moments of which are 1.596 and 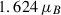, respectively) from those with magnetic moments of less than 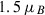 . The maximum local magnetic moment of LA-Co is 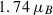. The leaf for which the local magnetic moment is largest, i.e. larger than 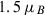, contains 36 positive and 19 negative cases, if we do not use (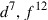) or (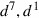). However, this leaf contains 34 positive and five negative cases if we use the information related to the T–LA interaction to cluster the cases appropriately.

For the case of the Mn sites, the trend is not as clear as for Fe, Co, or Ni. In fact, it is observed that the local magnetic moments for the Mn sites fall within a large range, i.e., from 0.0 to 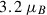. To obtain a local magnetic moment greater than 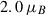, a (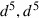) element less than 7.34 and (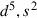) greater than 8.69 are required. However, among the 12 cases satisfying these conditions, only six positive cases were found. Further, three of the six negative cases exhibit local magnetic moments of less than 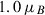, whereas the other three cases exhibit local magnetic moments of 1.0–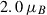. This observation can be attributed to the complex magnetic structures of the half-filled *d* orbitals.

These results show that clustering by the decision trees can determine important elements of the OFM that are consistent with the physical or chemical picture. Further, we can automatically derive quantitative relations between the elements of the OFM and the local magnetic moments using the developed method. Thus, we can expect that the OFM, which we employed as descriptors in the decision trees, can be good descriptors for the regression of the local magnetic moments of the LAT systems.

In the next step, we examine how the local magnetic moments can be represented by the OFM descriptors based on the fact that materials with higher similarity (as estimated by the descriptors) should possess similar local magnetic moments. For this purpose, we employ a simple nearest-neighbor regression method to predict the local magnetic moments, and the cross-validated root mean squared error (RMSE) is used to measure the performance of our descriptors. In the nearest-neighbor regression, a property of a data point is deduced from the properties of the nearest-neighbor points in the training data. For the quantitative prediction of physical properties, it is necessary to distinguish the valence orbital using a different principal quantum number, e.g., the 3*d* orbitals should differ from the 4*d* orbitals. Therefore, hereafter, we use Equation (2) with the distance weight to generate the descriptors for the local and global structures.

**Table 1.** Cross-validated RMSE (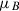) and 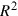 for predicted local magnetic moments obtained via nearest-neighbor regression with selected distance measurements (enumerated in the supplemental information).

Distance						
RMSE	0.26	0.21	0.23	0.21	0.21	0.23
	0.86	0.90	0.89	0.90	0.90	0.90

Table 1 summarizes the cross-validated RMSE and the coefficient of determination 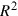 between the observed and predicted values obtained using our nearest-neighbor regression and different distance measurements. We achieve a reasonable performance as regards the prediction of the local magnetic moments, obtaining an RMSE of approximately 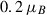 and an 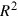 value of 0.9. This result indicates that close materials in our description space of a local structure yield similar local magnetic moments, which implies that our data representation includes significant information about the local magnetic moments. To further improve the prediction of the local magnetic moments, we apply KRR as the prediction model. We obtain a cross-validated RMSE of 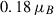, a cross-validated mean absolute error (MAE) of 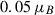, and an 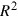 value of 0.93, as indicated in Table 2.

**Table 2.** Cross-validated RMSE (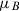), cross-validated MAE (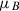), and 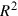 for predicted local magnetic moments obtained via KRR regression with OFM and CM descriptors.

Descriptor	OFM	CM
RMSE	0.18	0.21
MAE	0.05	0.11
R	0.93	0.90

To assess the capability of the OFM descriptor (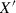 in Equation (2)), we compare its performance with that of the CM descriptor proposed by Rupp and coworkers [19–21]. We treat the local structures in the same manner as isolated molecules, and the calculated CM descriptors are used to predict the local magnetic moments using KRR regression. Using the CM descriptor, we obtain a cross-validated RMSE of approximately 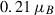, a cross-validated MAE of 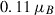, and an 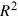 value of 0.90, as indicated in Table 2. The obtained results show that the OFM descriptor, which includes information on the coordination of valence electrons, is more informative and, consequently, yields a slight improvement in prediction accuracy compared to the CM descriptor for the local magnetic moments of the LAT alloys.

### 3.2. Prediction of material properties

With the aim of obtaining a prediction model with high prediction accuracy, the representation of materials is usually designed to include as much information as possible via a large number of descriptors, without considering their interpretability. In this work, as mentioned above, we focus on developing descriptors, taking both the applicability and interpretability into consideration. Therefore, instead of designing a complicated representation for materials, we choose a simple approach in which the descriptor of a material is derived by averaging or summing the descriptors for the local structures of its constituent atoms. Here, we implement the prediction models for the formation energies of crystalline systems and the atomization energies of molecular systems in order to examine the applicability of the OFM descriptors.

**Figure 3.**
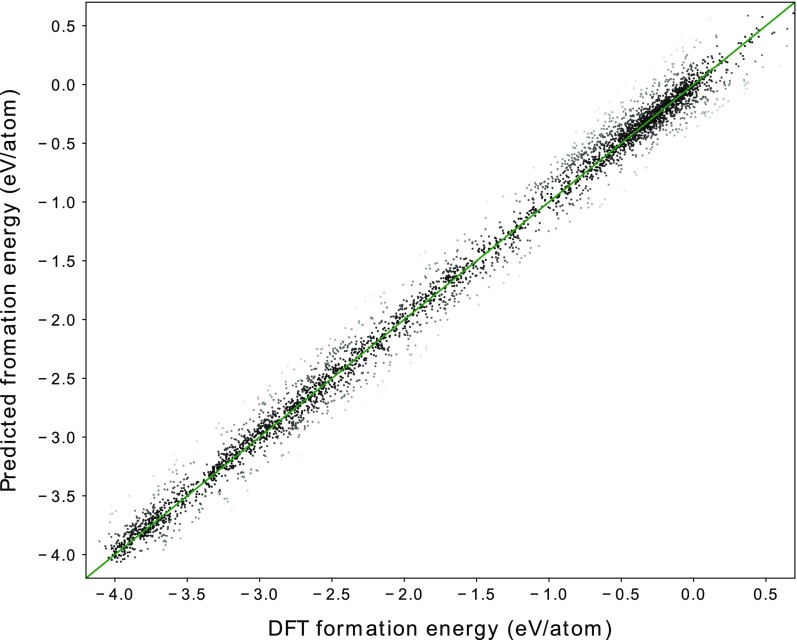
Comparison of formation energies calculated using DFT and those predicted through machine learning (ML-predicted), using OFM.

For crystalline systems, we focus on transition-metal binary alloys (TT), and bimetal alloys of lanthanide metal and transition-metal (LAT), as well as LATX and TTX, which are LAT and TT alloys that include a light element X. We select the transition-metals from the set of 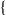
Sc, Ti, V, Cr, Mn, Fe, Co, Ni, Cu, Zn, Y, Zr, Nb, Mo, Tc, Ru, Rh, Pd, Ag, Cd, Hf, Ta, W, Re, Os, Ir, Pt, Au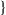, the lanthanides from 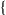
La, Ce, Pr, Nd, Pm, Sm, Eu, Gd, Tb, Dy, Ho, Er, Tm, Yb, Lu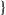, and the X elements from {B, C, N, O}. We collect the data of more than four thousand compounds, including their structures and formation energies, from the Materials Project repository: 1510 LATX compounds, 1311 TTX compounds, 692 LAT compounds, and 707 TT compounds. We use the average of the descriptors for their local structures to build the global descriptor for each of these materials.

For these crystalline systems, we compare the performance of our OFM descriptor (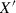 in Equation (4)) with that of the CM descriptor, which is based on the Ewald sum and which was developed by Faber and coworkers [20]. We use a KRR model with a Laplacian kernel for both the OFM and CM descriptors. A 10-fold cross-validated comparison between the DFT-calculated formation energies and the machine learning-predicted formation energies is shown for the OFM in Figure 3. The DFT-calculated and ML-predicted formation energies show good agreement, with an 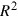 value of 0.98, a cross-validated RMSE of 0.19 eV/atom, and a cross-validated MAE of 0.11 eV/atom. This result is better than that obtained using the CM descriptor, which yields an 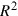 value of 0.87, a cross-validated RMSE of 0.47 eV/atom, and a cross-validated MAE of 0.39 eV/atom, as summarized in Table 3. A similar relatively poor result of the CM descriptor has been already reported on the performance in the prediction of the formation energies of crystal systems [20].

**Table 3.** Cross-validated RMSE (eV/atom), cross-validated MAE (eV/atom), and 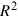 for formation energy of LATX and atomization energy of QM7 dataset obtained using OFM and CM descriptors.

Dataset	LATX		QM7	
Descriptor	OFM	CM [20]	OFM	CM [19]
RMSE	0.190	0.470	0.043	0.040
MAE	0.112	0.390	0.027	0.020
	0.98	0.87	0.98	0.99

For the molecular systems, we focus on the atomization energies of organic molecules. We use the QM7 dataset with 6915 organic molecules [19,40]. (Originally, the QM7 dataset contained 7195 molecules, but more than 100 molecules were removed because of a technical problem in determining Voronoi polyhedra for flat structures). As noted above, the descriptor of a molecule is built by summing over the descriptors of its local structures. Using our OFM representation, Equation (2), and KRR regression, we obtain a cross-validated RMSE of 0.043 eV/atom, a cross-validated MAE of 0.027 eV/atom, and an 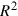 value of 0.98. In contrast, the CM yields a cross-validated RMSE of 0.040 eV/atom, a cross-validated MAE of 0.020 eV/atom, and an 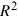 value of 0.99 [19–21], as indicated in Table 3. It is worth noting that although our dimension of our OFM seems to be high compared to CM for the small systems, the advantage of OFM is that its dimension is fixed regardless the size of the system. In fact, our OFM contains the only small number of non-zero elements depending on data set. For the QM7 data set, we only need 25 features.

**Figure 4.**
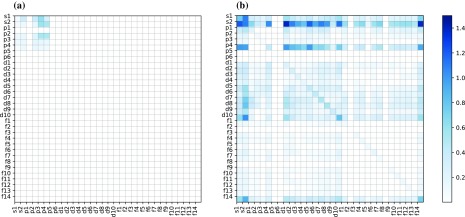
Standard deviations of local OFMs of QM7 (a) and LATX (b) datasets.

This result confirms that the construction of the OFM of a material, which is achieved by averaging or summing the descriptors of all the local structures of the constituent atoms, yields superior prediction accuracy than the CM descriptor for the formation energies of LATX systems, and comparable accuracy to the CM descriptor for the atomization energies of organic molecular systems in the QM7 dataset. It may be noted that, for molecular systems (the QM7 dataset contains light elements such as C, H, O, N, and S only), the CM descriptor yields a slightly better result than our OFM. However, for LATX systems with a variety of elements (the LATX dataset contains transition-metals, lanthanides, and light elements), our OFM exhibits superior prediction ability.

Figure 4(a) and (b) depicts the standard deviations of the OFMs of all local structures for the QM7 and LATX datasets, respectively. It is apparent that the QM7 dataset contains only a small number of non-zero OFM elements, whereas the LATX dataset exhibits a large variety of OFMs. Moreover, the QM7 dataset exhibits a small deviation of the OFM, whereas the LATX dataset has a greater deviation. These differences arise because the QM7 dataset is comprised of organic molecules, where the covalent bonding formed by the *sp*-pagination

hybridization is a major factor determining the geometry of the nearest neighbor atoms or the coordination number, in principle. Therefore, the QM7 dataset appears to be less divergent as regards the OFM descriptors. On the other hand, the crystalline materials in the LATX dataset, which includes so-called ionic bonding as well as covalent bonding, have considerably higher diversity in terms of both composition and structure. Interestingly, our OFM yields a better result for these complex and divergent systems. The important point to note is that the OFM can describe large diversity in atomic composition and structure more clearly, facilitating the learning and prediction of the properties of both crystalline and molecular systems. The OFM includes the effect of the nearest neighbor sites chosen by the Voronoi polyhedra only. However, for a molecule, the OFM can yield performance equivalent to that of the CM, the descriptors of which are based on long-range power-law decay. Further, the OFM results are of considerably better quality than those of the CM for the periodic LATX systems. Thus, our results indicate that our developed OFM technique offers an essential basis for the theoretical design of materials properties, via an approach similar to building blocks.

## 4. Conclusions

We have proposed a novel representation of crystalline materials named as ’orbital-field matrix (OFM)’, which is based on the distribution of valence shell electrons. We have demonstrated that this new representation can be highly useful in describing and measuring the similarities of materials or local structures in bimetal alloys of lanthanide metal and transition-metal (LAT) as well as LATX (X: light element) ternary alloys. Our experiments show that our OFM can accurately reproduce the DFT-calculated local magnetic moments of transition-metal sites in LAT alloys with a cross-validated RMSE of 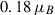 and an 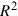 value of 0.93. Moreover, the results can be interpreted in the language of physical chemistry; that is, the ligand field theory for the local magnetic moment. Decision tree regression shows the importance of the coordination numbers of the occupied *d* orbitals of the transition-metals and the occupied *f* orbitals of the lanthanides in determining the local magnetic moments of the transition-metal sites. Further, the formation energies of crystalline systems and the atomization energies of molecular systems can be well predicted using our OFM. That is, with KRR representation, the formation energies of the crystalline systems and atomization energies of the molecular systems can be accurately reproduced with an 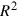 value of approximately 0.98. Incorporating information on the atomic orbital coordination, OFM exhibits superior applicability to systems with high diversity in atomic composition and structure in LATX compared to the CM approach. The acquired results suggest that OFM could be useful for mining chemical/physical information on materials from available datasets using modern machine learning algorithms.

Details of the methods and the model parameter optimization are summarized in the supplemental materials.

## Supplementary Material

Supplement.zip
