# Supplementary material for: Machine learning reveals orbital interaction in materials
Source: Sci Technol Adv Mater. 2017 Oct 26;18(1):756–65. doi: 10.1080/14686996.2017.1378060 (PMC5678453; doi:10.1080/14686996.2017.1378060)
Supplement: Supplement.zip [file TSTA_A_1378060_SM3016.zip › suppl.pdf]

SUPPLEMENTAL MATERIALS

I. ELECTRON CONFIGURATION

Z	element name	non-zero element(s) of one-hot-vector	atomic electron configuration
1	H	s^1	$1s^1$
5	B	s^2, p^1	$[He]2s^2 2p^1$
6	C	s^2, p^2	$[He]2s^2 2p^2$
7	N	s^2, p^3	$[He]2s^2 2p^3$
8	O	s^2, p^4	$[He]2s^2 2p^4$
16	S	s^2, p^4	$[Ne]3s^2 3p^4$
21	Sc	s^2, d^1	$[Ar]4s^2 3d^1$
22	Ti	s^2, d^2	$[Ar]4s^2 3d^2$
23	V	s^2, d^3	$[Ar]4s^2 3d^3$
24	Cr	s^1, d^5	$[Ar]4s^1 3d^5$
25	Mn	s^2, d^5	$[Ar]4s^2 3d^5$
26	Fe	s^2, d^6	$[Ar]4s^2 3d^6$
27	Co	s^2, d^7	$[Ar]4s^2 3d^7$
28	Ni	s^2, d^8	$[Ar]4s^2 3d^8$
29	Cu	s^1, d^{10}	$[Ar]4s^1 3d^{10}$
30	Zn	s^2, d^{10}	$[Ar]4s^2 3d^{10}$
39	Y	s^2, d^1	$[Kr]5s^2 4d^1$
40	Zr	s^2, d^2	$[Kr]5s^2 4d^2$
41	Nb	s^1, d^4	$[Kr]5s^1 4d^4$
42	Mo	s^1, d^5	$[Kr]5s^1 4d^5$
43	Tc	s^2, d^5	$[Kr]5s^2 4d^5$
44	Ru	s^1, d^7	$[Kr]5s^1 4d^7$
45	Rh	s^1, d^8	$[Kr]5s^1 4d^8$
46	Pd	d^{10}	$[Kr]4d^{10}$
47	Ag	s^1, d^{10}	$[Kr]5s^1 4d^{10}$
48	Cd	s^2, d^{10}	$[Kr]5s^2 4d^{10}$
57	La	s^2, d^1	$[Xe]6s^2 5d^1$
58	Ce	s^2, d^1, f^1	$[Xe]6s^2 5d^1 4f^1$
59	Pr	s^2, f^3	$[Xe]6s^2 4f^3$
60	Nd	s^2, f^4	$[Xe]6s^2 4f^4$
61	Pm	s^2, f^5	$[Xe]6s^2 4f^5$
62	Sm	s^2, f^6	$[Xe]6s^2 4f^6$
63	Eu	s^2, f^7	$[Xe]6s^2 4f^7$
64	Gd	s^2, d^1, f^7	$[Xe]6s^2 5d^1 4f^7$
65	Tb	s^2, f^9	$[Xe]6s^2 4f^9$
66	Dy	s^2, f^{10}	$[Xe]6s^2 4f^{10}$
67	Ho	s^2, f^{11}	$[Xe]6s^2 4f^{11}$
68	Er	s^2, f^{12}	$[Xe]6s^2 4f^{12}$
69	Tm	s^2, f^{13}	$[Xe]6s^2 4f^{13}$
70	Yb	s^2, f^{14}	$[Xe]6s^2 4f^{14}$
71	Lu	s^2, d^1, f^{14}	$[Xe]6s^2 5d^1 4f^{14}$
72	Hf	s^2, d^2, f^{14}	$[Xe]6s^2 5d^2 4f^{14}$
73	Ta	s^2, d^3, f^{14}	$[Xe]6s^2 5d^3 4f^{14}$
74	W	s^2, d^4, f^{14}	$[Xe]6s^2 5d^4 4f^{14}$
75	Re	s^2, d^5, f^{14}	$[Xe]6s^2 5d^5 4f^{14}$
76	Os	s^2, d^6, f^{14}	$[Xe]6s^2 5d^6 4f^{14}$
77	Ir	s^2, d^7, f^{14}	$[Xe]6s^2 5d^7 4f^{14}$
78	Pt	s^1, d^9, f^{14}	$[Xe]6s^1 5d^9 4f^{14}$
79	Au	s^1, d^{10}, f^{14}	$[Xe]6s^1 5d^{10} 4f^{14}$

II. SIMILARITY MEASURE

Once the representation for materials is derived, the next important step is the measurement of similarity (distance). In this work, we examine various types of distance measurements for measuring the similarity of local structures and materials represented by the proposed OFM: (1) Euclidean distance:

$$d_{eucl}(X,Y) = \sqrt{\sum_{i,j}(X_{ij} - Y_{ij})^2},$$

(2) Manhattan distance:

$$d_{man}(X,Y) = \sum_{i,j}|X_{ij} - Y_{ij}|,$$

(3) Cosine distance:

$$d_{cos}(X,Y) = 1 - \frac{\sum_{ij} X_{ij}Y_{ij}}{\sqrt{\sum_{i,j} X_{ij}^2}\sqrt{\sum_{i,j} Y_{ij}^2}},$$

(4) Bary-Curtis distance:

$$d_{bar}(X,Y) = \frac{\sum_{ij}|X_{ij} - Y_{ij}|}{\sum_{ij}|X_{ij}| + |Y_{ij}|},$$

(5) Canberra:

$$d_{can}(X,Y) = \sum_{ij}\frac{|X_{ij} - Y_{ij}|}{|X_{ij} + Y_{ij}|},$$

and (6) correlation:

$$d_{cor}(X,Y) = \frac{Cov(X,Y)}{\sqrt{\sigma_X \cdot \sigma_Y}}.$$

where $Cov(X,Y)$ is covariance between X and Y; σ_X and σ_Y are variance of X and Y, respectively.

III. MACHINE-LEARNING EXPERIMENTS

A. Decision tree

To learn the mechanism of determination of the local magnetic moment from data, we apply a decision tree regression model to represent the dependence of magnetic moment on the descriptors. The concept in the decision tree is that the prediction model is broken down into a set of choices for each descriptor element, in turn, starting at the root of the tree and progressing to the leaves, where the prediction result is received. The goal is to create a model that predicts the value of a target variable by learning simple and interpretable decision rules inferred from the data features [1]. We employ a decision tree builder using the variance of explanatory variables and tree pruning using reduced-error pruning with back fitting (REPTree) implemented in the Weka package [2].

TABLE I. Local magnetic moment discretization of transition metal elements for decision tree analysis.

Transition-metal element	Local magnetic moment		
	Low	Medium	High
Mn	$\leq 1.0\mu B$	$1.0\mu B \sim 2.0\mu B$	$\geq 2.0\mu B$
Fe	$\leq 2.2\mu B$	$2.2\mu B \sim 2.5\mu B$	$\geq 2.5\mu B$
Co	$\leq 0.5\mu B$	$0.5\mu B \sim 1.5\mu B$	$\geq 1.5\mu B$
Ni	$\leq 0.2\mu B$	$0.2\mu B \sim 0.4\mu B$	$\geq 0.4\mu B$

For a better interpretation, we discretized the range of the local magnetic moment of transition-metal sites into three levels: high, medium, and low. The discretization is carried out with considering the balance in the number of data instances in each level, as shown in Table 1. We performed ten fold cross-validation, and the structure of the decision trees appear to be stable.

B. Nearest-neighbor regression

The principle behind the nearest-neighbor methods is to find a predefined number of training samples at shortest distances to a new interest point and the predicted value of the target variable for this new point is weighted average over values of the target variable of its neighbors. The number of samples can be a user-defined constant (k-nearest-neighbor learning), or can vary based on the local density of points (radius-based neighbor learning). The accuracy of nearest-neighbor regression, therefore, indicates the performance of data representation and similarity measurement.

We employ a nearest-neighbor regressor implemented in the scikit-learn package [3]. The number of nearest neighbors is fixed as 5, and the nearest neighbors are determined by a brute-force search. The prediction is weighted by the distance to the nearest neighbors.

C. Kernel ridge regression

In the present work, the physical properties of materials are represented using kernel ridge regression (KRR)[4], which is a combination of the kernel method and ridge regression. This method has been used successfully in the recent past within the materials and chemical sciences. In the KRR algorithm, the property of a system can be given by the weighted kernel function:

$$y = f(x, c) = \sum_{k \in D_{ref}} c_k K(x, x_k), \tag{1}$$

where k runs over all the reference data (D_{ref}). We use Laplacian kernel function: $K(x, x_k) = e^{-\gamma d(x, x_k)}$, where $d(x, x_k)$ is the Euclidean distance function of (x, x_k). In order to minimize the prediction risk, the coefficients c_k are determined by minimizing the total square error regularized by L2 norm (ridge regression):

$$\arg\max_c \left(\sum_i [f(x_i, c) - y_i]^2 + \lambda \sum_k ||c_k||_2^2 \right). \tag{2}$$

Parameters γ and λ are determined in an inner loop of the 10-fold cross validation by using a logarithmic-scale grid to predict the local magnetic moment. We optimize the hyperparameters of the KRR model to predict formation energies, kernel width σ and regularization parameter λ, by minimizing the 10-fold cross-validated RMSE. The optimized parameters are identified by searching over 2500 pairs of σ and λ on a 2D logarithmic grid. These procedures are routinely applied in machine-learning and statistics to avoid overfitting and overly optimistic error estimates.

[1] S. Marsland, ed., *Machine learning: An Algorithmic perspective* (CRC Press, 2009).

[2] M. A. H. Eibe Frank and I. H. Witten, "The weka workbench. online appendix for "data mining: Practical machine learning tools and techniques", morgan kaufmann, fourth edition," (2016).

[3] F. Pedregosa, G. Varoquaux, A. Gramfort, V. Michel, B. Thirion, O. Grisel, M. Blondel, P. Prettenhofer, R. Weiss, V. Dubourg, J. Vanderplas, A. Passos, D. Cournapeau, M. Brucher, M. Perrot, and E. Duchesnay, Journal of Machine Learning Research **12**, 2825 (2011).

[4] K. P. Murphy, ed., *Machine learning: a probabilistic perspective* (MIT Press, 2012).
